# External Validation of European System for Cardiac Operative Risk Evaluation II (EuroSCORE II) for Risk Prioritization in an Iranian Population

**DOI:** 10.21470/1678-9741-2017-0030

**Published:** 2018

**Authors:** Alireza Atashi, Shahram Amini, Mohammad Abbasi Tashnizi, Ali Asghar Moeinipour, Mathias Hossain Aazami, Fariba Tohidnezhad, Erfan Ghasemi, Saeid Eslami

**Affiliations:** 1 Department of Medical Informatics, Faculty of Medicine, Mashhad University of Medical Sciences, Mashhad, Iran.; 2 Medical Informatics Department, Breast Cancer Research Center, Moatamed Cancer Institute, ACECR, Tehran, Iran.; 3 Department of Anesthesiology and Critical Care, Mashhad University of Medical Sciences, Mashhad, Iran.; 4 Department of Cardiac Surgery, Mashhad University of Medical Sciences, Mashhad, Iran.; 5 Cardiac Anesthesia Research Center, Mashhad University of Medical Sciences, Mashhad, Iran.; 6 Department of Biostatistics, School of Paramedical Sciences, Shahid Beheshti University of Medical Sciences, Tehran, Iran.; 7 Pharmaceutical Research Center, Mashhad University of Medical Sciences, Mashhad, Iran.; 8 Department of Medical Informatics, Academic Medical Center, University of Amsterdam, Amsterdam, the Netherlands.

**Keywords:** Mortality, Decision Support Techniques, Risk Assessment, Cardiac Surgical Procedures

## Abstract

**Introduction:**

The European System for Cardiac Operative Risk Evaluation II (EuroSCORE II) is a prediction model which maps 18 predictors to a 30-day post-operative risk of death concentrating on accurate stratification of candidate patients for cardiac surgery.

**Objective:**

The objective of this study was to determine the performance of the EuroSCORE II risk-analysis predictions among patients who underwent heart surgeries in one area of Iran.

**Methods:**

A retrospective cohort study was conducted to collect the required variables for all consecutive patients who underwent heart surgeries at Emam Reza hospital, Northeast Iran between 2014 and 2015. Univariate and multivariate analysis were performed to identify covariates which significantly contribute to higher EuroSCORE II in our population. External validation was performed by comparing the real and expected mortality using area under the receiver operating characteristic curve (AUC) for discrimination assessment. Also, Brier Score and Hosmer-Lemeshow goodness-of-fit test were used to show the overall performance and calibration level, respectively.

**Results:**

Two thousand five hundred eight one (59.6% males) were included. The observed mortality rate was 3.3%, but EuroSCORE II had a prediction of 4.7%. Although the overall performance was acceptable (Brier score=0.047), the model showed poor discriminatory power by AUC=0.667 (sensitivity=61.90, and specificity=66.24) and calibration (Hosmer-Lemeshow test, *P*<0.01).

**Conclusion:**

Our study showed that the EuroSCORE II discrimination power is less than optimal for outcome prediction and less accurate for resource allocation programs. It highlights the need for recalibration of this risk stratification tool aiming to improve post cardiac surgery outcome predictions in Iran.

**Table:** 

Abbreviations, acronyms & symbols	
AUC	= Area under the receiver operating characteristic curve
CABG	= Coronary artery bypass grafting
CCS	= Canadian Cardiovascular Society
EF	= Ejection factor
EuroSCORE	= European System for Cardiac Operative Risk Evaluation
NYHA	= New York Heart Association
RAMR	= Risk-adjusted mortality ratio
STS	= Society of Thoracic Surgeons

## INTRODUCTION

A growing literature shows the pervasiveness and importance of the need for reliable information on the cost-effectiveness of adult cardiac surgeries. Moreover, potential post-operative adverse events highlight the significance of perioperative clinical decision making. Various prediction models have been developed aiming to estimate risk-adjusted mortality, morbidity and length of intensive care unit stay following cardiac surgeries^[1]^. European System for Cardiac Operative Risk Evaluation (EuroSCORE) is a risk stratification tool which incorporates 18 variables describing patient, heart and proposed surgery to predict 30-day post operative risk of death^[2]^. Predictive power of EuroSCORE II has been evaluated on different samples of target population in European countries. Vast majority of these studies have reported acceptable calibration (How many patients with a risk prediction of x% have experienced the outcome?) and discrimination (Who are the patients who have experienced the outcome associated with higher risk predictions and who are those that do not?) measures in comparison to Society of Thoracic Surgeons (STS) [esp. for patients undergoing coronary artery bypass grafting (CABG) procedure^[3]^].

An international evaluation study was performed by Roques et al.^[4]^, in 2000, to assess the predictive ability of EuroSCORE II on 18676 patients from six European countries (Germany, Spain, England, France, Italy, and Finland). Despite clinical and epidemiological differences, EuroSCORE II provided acceptable predictions for all datasets (esp. for Spanish patients). Geissler et al.^[5]^ compared six prediction models using a single-center 2-year dataset, which resulted in the best performance measures for EuroSCORE II. While previous studies published admissible application of EuroSCORE II for patients undergoing CABG^[6,7]^, conflict reports exist for Australian samples^[8]^.

Similar studies in Iran reflect poor applicability of EuroSCORE II within patients undergoing different types of cardiac surgeries^[9,10]^. Diverse surgical techniques and potential risk factors already have been stabilized in different communities may mislead prediction models and result in erroneous interpretations. Thus, mathematical localization studies are required in different geographical borders to assure its proper predictive function before routine clinical use^[11]^. This study is conducted to investigate the accuracy of quantitative prioritization scores estimated by EuroSCORE II in an Iranian population.

## METHODS

### Participants and Setting

A retrospective single-center cohort study was conducted to include all consecutive patients undergoing cardiac surgeries at Emam Reza hospital, Northeast Iran from January 1, 2014 to December 31, 2015. Once the patient was hospitalized a cardiologist or a general physician evaluated pre- peri- and postoperative state to fill out the pre-designed structural paper form.

A total of 2907 patients were included and 30-day outcome was discovered using hospital information system or direct contact with patients' family. About 11.2% (N=326) of records were excluded due to major variables' missing values and all data items were rechecked to verify their consistency, reliability and integrity. In some cases (less than 3% of records) by the physicians' recommendation, the missing data were imputed with normal values.

### Statistical Analysis

First, univariate and multivariate analysis of relevant EuroSCORE II prognostic factors were performed aiming to identify significant covariates which contributed to higher risk. EuroSCORE II was calculated and inserted in dataset using online calculator (Available at: http://riskcalc.sts.org/stswebriskcalc/#). The data were aggregated in a unique electronic dataset, summarized considering the demographic and clinical characteristics and were used for statistical analysis. The relation of each variable was addressed and the number of patients due to different values were compared to the original EuroSCORE II population. Then, the overall model performance was reported using Brier Score (A score function which measures the closeness of predictions to actual outcomes and result in a value from 0 for a perfect model to 0.25 for a non-informative model)^[12]^. The area under the receiver operating characteristic curve (AUC) statistic was used to indicate the discriminative ability of model (while 1 refers to perfect discrimination, a value of 0.5 shows random classification). The Hosmer-Lemeshow goodness-of-fit test was employed to test the fitness of model to data by comparing observed to predicted mortality by decile of predicted probability)^[13]^. Analysis were performed using Medcalc-13.3.3.0 and R-3.3.1 (Resource Selection package).

## RESULTS

### Patients' Baseline Characteristics

The mean age among the total of 2581 patients was 56.3±13.88 years (minimum=17 and maximum=93). The mortality rate was 3.3% (N=84). The mean height and weight of patients were 1.64±0.1 meters and 68.4±13.4 kilograms, respectively. About 7.8% (N=201) of patients aged 75 years and older and 22.2% (N=572) were diabetic. While 6.1% (N=158) were involved with a type of chronic kidney disease, 15.1% (N=24) underwent dialysis regularly; 10.6% (N=274) were current or past smokers and 2.2% (N=56) of patients were diagnosed with COPD. Table 1 summarizes some comparable information of our patients with the original EuroSCORE II population.

**Table 1:** Comparison of demographic and comorbidity characteristics between the original EuroSCORE II population and an Iranian sample^[2]^.

Variable		Frequencies (%) or mean (SD) [range] of original EuroSCORE II Population (N=22381)	Frequencies (%) or mean (SD) [range] of our Population (N=2581)
Age		64.6 (12.5) [18-95]	56.3 (13.88) [17-94]
Gender	Female	6919 (30.9%)	1044 (40.4%)
	Male	15462 (69.1%)	1537 (59.6%)
Height (cm)		168.5 (9.6) [100-213]	164.1 (10.0) [104-199]
Weight (kg)		77.9 (15.9) [30-182]	68.4 (13.4) [28-132]
BMI (kg/m^2^)		27.4 (4.8) [9.6-82.6]	25.4 (4.8) [10.1-62.6]
Diabetes on insulin		5643 (25.2%)	572 (22.2%)
NYHA	Class II	NA	1008 (37.0%)
	Class III	NA	957 (37.1%)
	Class IV	NA	96 (3.6%)
Chronic pulmonary disease		2384 (10.7%)	56 (2.2%)
Serum creatinine (mg/dl)		1.13 (0.92)	1.09 (0.98)
Renal failure		108 (0.5%)	158 (6.1%)
	Dialysis		23 (0.9%)
LV function (ejection fraction)	EF≤50	NA	1150 (44.6%)
	51-70	NA	782 (30.3%)
	EF≤70	NA	20 (0.8%)
Recent MI		NA	161 (6.2%)
Pulmonary hypertension		NA	190 (7.3%)
Previous cardiac surgery		NA	8 (0.3%)
Urgency	Urgent operation	4135 (18.5%)	None
	Emergency	972 (4.3%)	None
	Elective	17 165 (76.7%)	2581 (100%)
	Salvage	109 (0.5%)	None

NA=not available; BMI=body mass index; NYHA=New York Heart Association functional classification; LV=left ventricle; MI=myocardial infarction

As all procedures were elective operations, there were no urgent surgeries. Also, 23 patients undergoing valve surgery were suffering from active endocarditis, extra cardiac arteriopathy. Poor mobility was observed in 48 patients. No patient with Canadian Cardiovascular Society (CCS) class 4 or with critical preoperative state was observed. Also, none of surgeries were on thoracic aorta. Some other details are presented in Tables 1 and 2. As these patients had no mortality, these factors were excluded for regression analysis. The univariate and multivariate analysis are presented in Table 3.

**Table 2:** EuroSCORE II characteristics by patients' demographic and clinical characteristics; number of patients and relation with mortality. The expected and observed mortality can also be compared by any variable^[2]^.

	Variable	Number of Patients N (%)	Mortality N (%)	Mortality Predicted truly (N) by EuroSCORE II
Gender	Male	1537 (59.6%)	40 (47.6%)	27
	Female	1044 (40.4%)	44 (52.4%)	29
Age	≤20	35 (1.4%)	__	__
	21-40	301 (11.7%)	6 (7.1%)	3
	41-60	1215 (47.1%)	30 (35.7%)	21
	61-80	979 (37.9%)	45 (53.6%)	31
	>80	46 (1.8%)	3 (3.6%)	1
BMI (kg/m^2^)	≤18.5	120 (4.6%)	10 (11.9%)	8
	[18.5-23]	608 (23.6%)	21 (25%)	14
	[23-25]	437 (16.9%)	10 (11.9%)	8
	[25-30]	832 (32.2%)	28 (33.4%)	21
	>30	364 (14.1%)	7 (8.4%)	5
Valve Surgery (weight of the intervention)	Isolated CABG	2071 (80.2%)	54 (64.3%)	37
	AVR	76 (2.9%)	2 (2.4%)	2
	MVR	195 (7.6%)	14 (16.7%)	11
	TVR	16 (0.6%)	2 (2.4%)	2
	MVR+TVR	54 (2.1%)	4 (4.7%)	1
	AVR+MVR	47 (1.8%)	3 (3.6%)	2
	ASD+TVR	10 (0.4%)	__	__
	ASD	7 (0.3%)	__	__
	AVR+MVR+TVR	18 (0.7%)	1 (1.2%)	__
	PVR	10 (0.4%)	__	__
	2 procedures	122 (4.7%)	7 (8.3%)	6
	3 procedures	19 (0.7%)	1 (1.2%)	__
	Other	77 (3%)	4 (4.7%)	3
Ejection fraction	≤50	1150 (44.6%)	64 (76.1%)	31
	51-70	782 (30.3%)	19 (22.6%)	10
	>70	20 (0.8%)	1 (1.2%)	1
Diabetes mellitus	Yes	572 (22.2%)	32 (38.1%)	19
	No	2009 (77.8%)	52 (61.9%)	37
COPD	Yes	56 (2.2%)	3 (3.6%)	3
	No	2525 (97.8%)	81 (96.4%)	51
Mortality	Alive	2497 (96.7%)	N/A	N/A
	Dead	84 (3.3%)	N/A	56
Previous cardiac surgery	Yes	8 (0.3%)	3 (3.6%)	1
	No	2573 (99.7%)	81 (96.4%)	55
Recent MI	Yes	161 (6.2%)	7 (8.3%)	4
	No	2420 (93.8%)	77 (91.7%)	52
NYHA	Class II	1008 (37.0%)	7 (8.3%)	2
	Class III	957 (37.1%)	24 (28.5%)	20
	Class IV	96(3.6%)	4 (4.7%)	4
Renal failure	Yes	158 (6.1%)	14 (16.6%)	10
	No	2423 (93.9%)	70 (83.3%)	46
Dialysis	Yes	23 (0.9%)	2 (2.4%)	2
	No	2558 (99.1%)	82 (97.6%)	54
Pulmonary hypertension	Yes	190 (7.3%)	15 (17.9%)	10
	No	2391 (92.6%)	64 (82.1%)	46

BMI=body mass index; CABG=coronary artery bypass grafting; AVR=aortic valve replacement; MVR=mitral valve replacement; TVR=tricuspid valve replacement; ASD=atrial septal defect; PVR=pulmonary valve replacement; COPD=chronic obstructive pulmonary disease; N/A=not applicable; MI=myocardial infarction

^a^Analysis by independent-samples t test.

^b^Analysis by one-way ANOVA.

Sum of percentages may not result in 100% due to missing data.

**Table 3:** Univariate and multivariate analysis of EuroSCORE II prognostic factors[2].

Characteristic		Mean ± SD	Univariate Analysis		Multivariate Analysis^a^	
			β (95% CI)	*P* value	β (95% CI)	*P* value
Age (year)^a^		56.3±13.88	0.063 (0.058 to 0.068)	<0.001	0.058 (0.051 to 0.065)	<0.001
Gender	Female	3.5±1.95	1 [References]	<0.001	1 [Reference]	
	Male	2.6±2.16	-0.88 (-1.05 to -0.72)		-0.82 (-0.98 to -0.66)	<0.001
Creatinine clearance	<50	4.4±2.16	1 [References]	<0.001	1 [Reference]	
	50-85	2.9±1.99	-1.49 (-1.70 to -1.28)		-0.92 (-1.13 to -0.71)	<0.001
	>85	1.8±1.53	-2.57 (-2.8 to -2.34)		-1.06 (-1.32 to -0.79)	<0.001
	Dialysis	3.0±2.31	-1.33 (-2.12 to -0.55)		-0.78 (-1.73 to -0.02)	0.045
Chronic lung disease	No	2.9±2.11	1 [References]	0.061	1 [Reference]	
	Yes	3.5±2.43	0.54 (-0.03 to 1.10)		0.15 (-0.31 to 0.61)	0.529
Diabetes on insulin	No	3.0±2.12	1 [References]	0.859	1 [Reference]	
	Yes	2.9±2.14	-0.02 (-0.22 to 0.18)		-0.19 (-0.37 to -0.01)	0.043
NYHA	Class I	2.4±1.97	1 [References]	<0.001	1 [Reference]	
	Class II	2.9±2.18	0.47 (0.18 to 0.77)		0.2 (-0.002 to 0.40)	0.052
	Class III	3.3±2.09	0.86 (0.56 to 1.17)		0.14 (-0.07 to 0.35)	0.199
	Class IV	3.8±1.98	1.44 (0.81 to 2.07)		0.52 (0.08 to 0.95)	0.019
Left ventricular function	≤50	3.5±2.19	1 [References]	<0.001	1 [Reference]	
	51-70	2.3±1.84	-1.22 (-1.40 to -1.03)		-0.87 (-1.03 to -0.70)	<0.001
	>70	2.0 ± 1.76	-1.52 (-2.43 to -0.61)		-1.37 (-2.06 to -0.69)	<.001
Recent myocardial infarction	No	2.8±2.07	1 [References]	<0.001	1 [Reference]	
	Yes	4.7±2.19	1.81 (1.48 to 2.14)		1.86 (1.56 to 2.15)	<0.001
Pulmonary hypertension	No	2.8±2.03	1 [References]	<0.001	1 [Reference]	
	Yes	5.2±1.99	2.41 (2.11 to 2.71)		2.04 (1.77 to 2.31)	<0.001
Weight of the intervention	Isolated CABG	2.7±2.06	1 [References]	<0.001	1 [Reference]	
	Single non-CABG	4.1±2.02	1.38 (1.14 to 1.62)		1.48 (1.25 to 1.72)	<0.001
	2 Procedures	4.2±1.93	1.55 (1.21 to 1.90)		1.89 (1.56 to 2.22)	<0.001
	3 Procedures	4.1±1.6	1.45 (0.74 to 2.15)		1.58 (0.91 to 2.25)	<0.001

a Using linear regression, all variables were found to be associated with EuroSCORE II, except for chronic lung disease, diabetes on insulin, and NYHA.

NYHA=New York Heart Association; CABG=coronary artery bypass graft

### Patients' Heart Status

Using New York Heart Association (NYHA), 37.1% (N=957) were classified as stage III cardiac failure patients, 6.2% (N=161) patients had a previous congestive heart failure during three months before surgery, 1% (N=26) of patients had atrial fibrillation. While 61.4% of surgeries were on-pump, the rest of procedures were performed off-pump. Table 2 shows more information about biological and clinical characteristics of patients.

### Performance Measures

As mentioned before, the overall mortality was 3.3%. When applied to the current data set, the EuroSCORE II predicted a mortality of 4.7%. This means that the current risk-adjusted mortality ratio (RAMR=observed/predicted) for the previous additive model is about 0.67 and not adequately enough for outcome prediction or resource allocation programs.

**Fig. 1:**
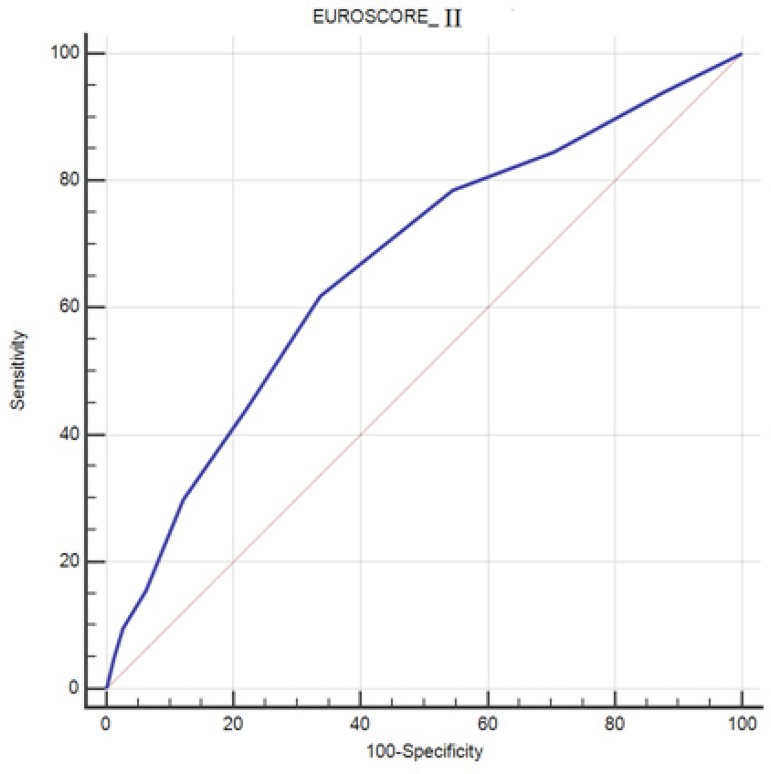
Area under the ROC curve for EuroSCORE II.

The Brier Score lower than 0.05 indicates acceptable overall performance. However, poor discrimination may be revealed by AUC=0.667 (cut off=3.0, sensitivity=61.90, and specificity=66.24). Also, the Hosmer-Lemeshow test showed unacceptable matching of predicted probabilities to observed events (*P*-value<0.01) (Table 4). Performance measures of EuroSCORE II are presented in Figure 1 and Table 4.

**Table 4:** Performance measures calculated for EuroSCORE II scoring system.

Scoring System	Overall Performance	Discrimination					Calibration
	Brier Score (min-max) [STD]	AUC	SE	95% CI	Sensitivity	Specificity	H-L Test
EuroSCORE II	0.047 (0.0-1.0) [0.12]	0.667	0.0307	0.648-0.685	0.619	0.662	Chi^2^(8)=936.66, *P*<0.01

AUC=area under the ROC curve; SE=standard error; CI=confidence interval, H-L=Hosmer-Lemeshow

## DISCUSSION

### Main Finding

Our single-center study, based on consecutive patients who underwent cardiac surgery revealed that EuroSCORE II demonstrated a moderate statistical overall performance with poor discrimination and calibration measures remain as concerning issues regarding 30-day post-operative mortality prediction after adult cardiac surgery. The analysis of ROC curve showed that the EuroSCORE II discrimination power is less than optimal (AUC=0.667) for outcome prediction and less accurate for resource allocation programs, because, references consider an AUC value more than 0.7 as an acceptable value for least useful prediction models^[5]^. Although, the Brier score less than 0.05 indicates good overall performance for the model^[12]^, the Hosmer-Lemeshow test showed unacceptable matching of predicted probabilities to observed events. In general, EuroSCORE II did not predict the outcome for our population as well as it did for the European populations. Thus, recalibration process seems to be essential for Iranian population prior to daily clinical use.

It is well known that risk assessment is central in the evaluation of the perioperative risk. The application of risk stratification tools gives an objective appraisal of risk for both physicians and patients and presents a good estimation for allocation of resources. However, some features may not be fully covered by the models including center-to-center variability in outcomes, type of surgery required and inherent complexity of some diseases^[14]^. On the other hand, the patient populations defer significantly between institutions and countries. Thus, the comparison of absolute numbers such as the mortality rates is not feasible. A large variety of risk scores have been developed due to differences in patient populations and comparisons of observed mortality versus expected mortality have been reported^[15-17]^.

### Comparison to Similar Studies

EuroSCORE was first developed in 1999 to estimate postsurgical mortality in a European population who underwent cardiac surgery (70% of procedures were CABG)^[6]^. A meta-analysis by Parolari et al.^[18]^ revealed poor performance of EuroSCORE II in valve surgery, in 2010. Moreover, as the quality of medical techniques continues to improve over time, model expiration was considered as an inevitable topic in this area ^[2,18-20]^. To address the surgical type bias in the modified version of model, reasonable number of patients who experienced CABG and valve surgeries were included in the development dataset^[2]^. The new version of EuroSCORE reflected acceptable discrimination power among both European and non-European samples^[17,21-23]^. The new model also provided more acceptable predictions for surgeries other than CABG^[21-23]^. However, evaluation studies in Iran reported poor performance measures for both EuroSCORE I and II^[9,10]^. Further studies concentrating on recalibrated version of the model published unacceptable results^[2,15,21,23]^.

The observed 30-day mortality rate in our sample (3.3%) was similar to those published by Roques et al.^[7]^ (3.4%), Nashef et al.^[6]^ (3.9%), Geissler et al.^[5]^ (4%), and Pitkänen et al.^[1]^ (2%). While Mir Mohammad Sadeghi et al.^[9]^ reported similar mortality rate in Isfahan (central Iran), four years later Jamaati et al.^[10]^ evaluated EuroSCORE II on a sample containing 12.2 mortality rate. An AUC of 66.7% in our study is lower than all similar studies including 78% by Geissler et al.^[5]^, 77% by Pitkänen et al.^[1]^, and 75.4% by Antunes et al.^[24]^. This is while similar studies in Iran confirmed the poor discriminative ability of EuroSCORE II^[9,10]^.

Currently, a great interest for prediction models as powerful tools for outcome prediction, cost-effectiveness strategies, reasonable resource allocation, and consequently quality control process have been growing^[1,9,25]^.

Due to the results of our study, despite the little differences between two populations (Tables 1 to 3) the EuroSCORE II may not be completely reliable for risk periodization or resource allocation programs in Iran. Poor performance measures for EuroSCORE II highlights the need for reformulating this risk stratification tool aiming to improve post cardiac surgery outcome predictions in Iran. It may be done by calibrating mortality risk scoring model (*e.g.* EuroSCORE model) for the region or creating new models with accurate localized parameter sets^[11,20]^.

### Limitation

Although sampling was done in one of the largest hospitals performing various cardiac procedures and the study has adequate sample size, including just one center may affect the generalizability of results to the entire country.

### Future Studies

Regarding the key prognostic role of prediction models, further investigation of clinical risk factors and recalibration process seems to be essential on large samples of target population from different centers around country aiming to improve outcome predictions.

## CONCLUSION

Our study showed that the EuroSCORE II discrimination power is less than optimal for outcome prediction and less accurate for resource allocation programs. It highlights the need for recalibration this risk stratification tool aiming to improve post cardiac surgery outcome predictions in Iran.

**Table:** 

Authors' roles & responsibilities	
AA	Substantial contributions to the conception or design of the work; or the acquisition, analysis, or interpretation of data for the work; drafting the work; final approval of the version to be published
SA	Substantial contributions to the conception or design of the work; or the acquisition, analysis, or interpretation of data for the work; data gathering management; final approval of the version to be published
MAT	Substantial contributions to the conception or design of the work; or the acquisition, analysis, or interpretation of data for the work; data gathering management; final approval of the version to be published
AAM	Substantial contributions to the conception or design of the work; or the acquisition, analysis, or interpretation of data for the work; data gathering management; final approval of the version to be published
MHA	Substantial contributions to the conception or design of the work; or the acquisition, analysis, or interpretation of data for the work; data gathering management; final approval of the version to be published
FT	Statistical analysis and interpretation of data for the work; final approval of the version to be published
EG	Statistical analysis and interpretation of data for the work; final approval of the version to be published
SE	Substantial contributions to the conception or design of the work; or the acquisition, analysis, or interpretation of data for the work; data gathering management; final approval of the version to be published
